# Unraveling the hypoxia modulating potential of VEGF family genes in pan-cancer

**DOI:** 10.5808/gi.23061

**Published:** 2023-09-27

**Authors:** So-Hyun Bae, Taewon Hwang, Mi-Ryung Han

**Affiliations:** 1 Division of Life Sciences, College of Life Sciences and Bioengineering, Incheon National University, Incheon 22012, Korea; 2 Department of Electrical and Electronic Engineering, Yonsei University, Seoul 03722, Korea

**Keywords:** angiogenesis, pan-cancer, tumor hypoxia, VEGF family genes

## Abstract

Tumor hypoxia, oxygen deprivation state, occurs in most cancers and promotes angiogenesis, enhancing the potential for metastasis. The vascular endothelial growth factor (VEGF) family genes play crucial roles in tumorigenesis by promoting angiogenesis. To investigate the malignant processes triggered by hypoxia-induced angiogenesis across pan-cancers, we comprehensively analyzed the relationships between the expression of VEGF family genes and hypoxic microenvironment based on integrated bioinformatics methods. Our results suggest that the expression of VEGF family genes differs significantly among various cancers, highlighting their heterogeneity effect on human cancers. Across the 33 cancers, *VEGFB* and *VEGFD* showed the highest and lowest expression levels, respectively. The survival analysis showed that *VEGFA* and placental growth factor (*PGF*) were correlated with poor prognosis in many cancers, including kidney renal cell and liver hepatocellular carcinoma. *VEGFC* expression was positively correlated with glioma and stomach cancer. *VEGFA* and *PGF* showed distinct positive correlations with hypoxia scores in most cancers, indicating a potential correlation with tumor aggressiveness. The expression of miRNAs targeting VEGF family genes, including hsa-miR-130b-5p and hsa-miR-940, was positively correlated with hypoxia. In immune subtypes analysis, *VEGFC* was highly expressed in C3 (inflammatory) and C6 (transforming growth factor β dominant) across various cancers, indicating its potential role as a tumor promotor. *VEGFC* expression exhibited positive correlations with immune infiltration scores, suggesting low tumor purity. High expression of *VEGFA* and *VEGFC* showed favorable responses to various drugs, including BLU-667, which abrogates RET signaling, an oncogenic driver in liver and thyroid cancers. Our findings suggest potential roles of VEGF family genes in malignant processes related with hypoxia-induced angiogenesis.

## Introduction

Hypoxia is a state of oxygen deficiency commonly observed in most cancers. Tumor hypoxia causes the formation of blood vessels, enhancing the potential for metastasis. Previous studies revealed that hypoxia strongly induces vascular endothelial growth factor (VEGF) expression [1]. Angiogenesis is the process of creating new blood vessels from pre-existing vessels. Through this process, cells receive blood containing oxygen and nutrients for growth. Moreover, as the degree of angiogenesis increases in primary tumors, the prognosis worsens [2]. VEGF-VEGF receptors, well-known as key angiogenic factors, have been shown to play crucial roles in tumor initiation, progression, and metastasis. Despite ongoing efforts to investigate association between hypoxia and genes that promote angiogenesis, no studies have been conducted using VEGF family genes across pan-cancer.

The VEGF family comprises *VEGFA*, *VEGFB*, *VEGFC*, *VEGFD*, and placental growth factor (*PGF*), and they differ in function and expression. For a few cancers, overexpression of VEGF family genes and their correlation with the prognosis, metastasis, and recurrence have been reported. Compared to low expression, high *VEGFA* expression is associated with poor survival outcomes in gastric cancer [3], lung cancer [4], and colon cancer [5]. *VEGFB* facilitates tumor advancement by elevating plasminogen activators, which can lead to the metastasis of breast cancer [6]. The expression of *VEGFC* and *VEGFD* correlates with recurrence in head and neck squamous cell carcinomas [7] and lymphatic metastases in gastric cancer [8], respectively. Previous study reported that up-regulated *PGF* is associated with lymph node metastases and serosal invasion in gastric cancer [9].

Despite the potential involvement of the VEGF family genes in various tumor-related pathways, little is known of their roles in the tumor microenvironment (TME) and their impact on tumor hypoxia across pan-cancer. Therefore, the link between expression of the VEGF family genes and hypoxia, prognoses, immune subtypes, and pharmacological activity should be explored. Here, we comprehensively analyzed the expression patterns of VEGF family genes and their association with hypoxia scores, survival rates, immune subtypes, TME, and responses to chemotherapy in 33 cancers.

## Methods

### Data collection

Pan-cancer data were downloaded (September 2022) from the UCSC Xena website (http://xena.ucsc.edu/). RNA-sequencing gene expression data (HTSeq-FPKM), micro-RNA (miRNA) expression data, survival data, clinical data, and immune subtypes were obtained from the Cancer Genome Atlas (TCGA) database. Data from TCGA comprised 11,057 samples from 33 cancers. The TCGA abbreviations and the detailed sample information, including tumor stage, and the number of tumor and normal samples, were summarized in Supplementary Table 1.

### Gene expression analysis

The average expression of VEGF family genes was estimated in 33 cancers with average of each cancer type. To compare the expression of VEGF family genes between tumors and normal tissues, we performed the Wilcoxon signed-rank test on 18 cancers which have more than five normal samples. The p-value was adjusted with Benjamini-Hochberg method and adjusted p-value < 0.05 was considered as significant. We estimated the expression correlation between the VEGF family genes using Spearman’s correlation method.

### Survival and tumor stage analysis

The patients were divided into two groups, high expression and low expression, based on the median expression level of each VEGF family gene. To analyze the prognostic differences between two groups, we used the “survival” and “survminer” R package to draw the Kaplan-Meier survival curves. The log-rank test was used to assess significance at p < 0.05. We explored the association between the expression of VEGF family genes and overall survival using univariate Cox proportional hazards regression. Moreover, the association between tumor stage and the expression of VEGF family genes was analyzed using the Kruskal-Wallis test and Benjamini-Hochberg p-value correction. Adjusted p-value < 0.05 was considered as significant.

### Hypoxia analysis

Tumor hypoxia was quantified using published gene signatures in 20 cancers [10]. The mRNA data of the genes in the hypoxia signature were extracted from each cancer and combined as a single cohort to compare hypoxia across cancers. According to Bhandari et al. [10], cancers with mRNA abundance values in the top 50% for each gene signature were assigned a score of +1. Cancers with mRNA abundance values in the bottom 50% for that gene were assigned a score of –1. Using the Spearman correlation method, we demo*nst*rated an association between hypoxia and the expression of VEGF family genes and miRNAs targeting them from all tumor samples of each cancer type.

### Immune and TME analysis

Six immune subtypes, namely C1 (wound healing), C2 (interferon-γ dominant), C3 (inflammatory), C4 (lymphocyte-depleted), C5 (immunologically quiet), and C6 (transforming growth factor β [TGF-β] dominant), were identified using the global transcriptomic immune classification of solid tumors [11]. Because each immune subtype has various clinical and biological characteristics, understanding how the expression of VEGF family genes relates to the immune subtype affects cancer treatment determination is important. To analyze the relationship between the expression of VEGF family genes and the six immune subtypes, we performed differential expression analysis by using the Kruskal-Wallis test and Benjamini-Hochberg adjustment. Adjusted p < 0.05 indicated statistical significance.

To investigate the TME which consists of tumor cells and normal cells, such as stromal and immune, the ESTIMATE algorithm and Spearman’s correlation method were used to examine correlation between tumor purity and the expression of VEGF family genes from all tumor samples of each cancer type. By the ESTIMATE algorithm [12], the gene expression levels of a specific sample were normalized based on their ranks, and an enrichment score was generated by summing the difference of the empirical cumulative distribution functions (CDFs) of the signature genes and those of the remaining non-signature genes according to [13]. For a given signature *S* with *n_S_* genes and a single sample *T*, the *n* genes in the data set are assigned ranks based on their absolute expression levels and ordered by their rank from highest (*nst*) to lowest (1_st_), represented as *L* = {*r*_1_, *r*_2_, ..., *r_n_*}. An enrichment score *ES*(*S*, *T*) is obtained by summing the difference between the weighted empirical CDF of the signature genes $P_{S}^{w}$ and the empirical CDF of the non-signature genes *P_n_S__*. When calculating the $P_{S}^{w}$, *α* was set to 0.25 to apply an appropriate weight to the ranks.


$$ES\left(S,T\right)=\;\overset{n}{\underset{i=1}{\sum }}[P_{S}^{w}\left(S,T,i\right)-P_{n_{S}}\left(S,T,i\right)]$$

where $P_{S}^{w}\;(S,\;T,\;i)\;=\;\sum_{r_{j}\in S,\;j\leq 1}^{}\frac{{\left|r_{j}\right|}^{\alpha }}{\sum_{r_{j}\in S{\left|r_{j}\right|}^{\alpha }}^{}}$ and $P_{n_{S}}(S,\;T,\;i)\;=\;\Sigma_{r_{j}\notin S,\;j\leq i}\frac{1}{\left(n-n_{S}\right)}$

### Drug sensitivity analysis

Transcripts and compound activity data were downloaded from the CellMiner database (https://discover.nci.nih.gov/cellminer/) that contains 60 human cancer cell lines (NCI-60). Raw data were processed using “impute” R package. Pearson’s correlation analysis was used to explore the correlation between the expression of VEGF family genes and drug sensitivity. Drugs used in clinical trials and Food and Drug Administration–approved drugs were included.

## Results

### Expression patterns of VEGF family genes

**Fig. 1.**
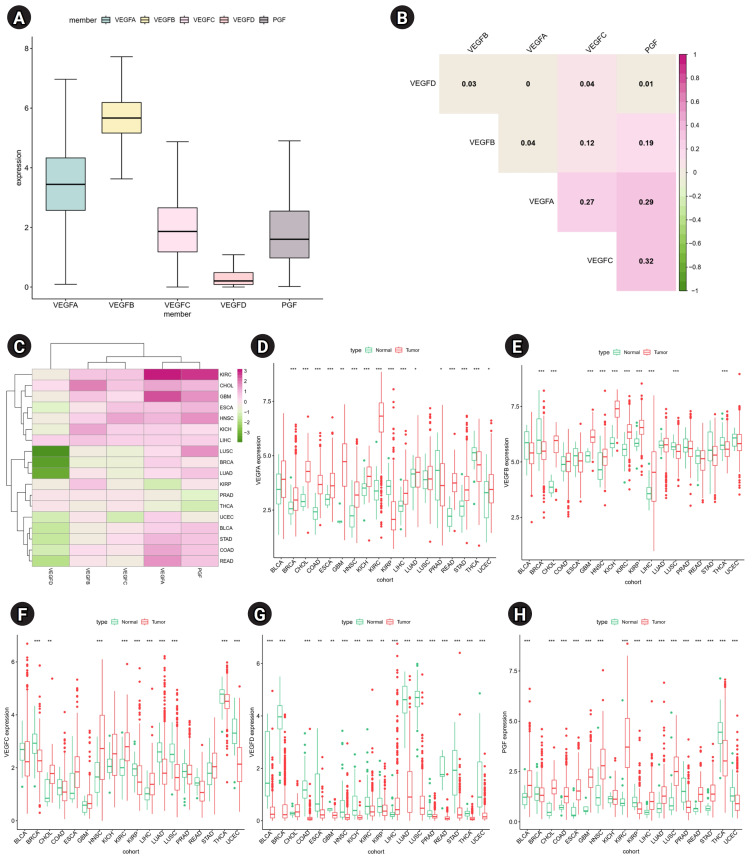
Expression patterns of vascular endothelial growth factor (VEGF) family genes. (A) Distribution of expression level of VEGF family genes across 33 cancers. (B) Associations between the expression of individual VEGF family genes. Pink and green indicate positive and negative correlations, respectively. (C) The differences in mean expression level of VEGF family genes between tumor and normal tissues. *VEGFA* (D), *VEGFB*, (E), *VEGFC* (F), *VEGFD* (G), and placental growth factor *(PGF)* (H) gene’s differential expression levels between tumor and normal tissues. BLCA, bladder urothelial carcinoma; BRCA, breast invasive carcinoma; CHOL, cholangiocarcinoma; COAD, colon adenocarcinoma; ESCA, esophageal carcinoma; GBM, glioblastoma multiforme; HNSC, head and neck squamous cell carcinoma; KICH, kidney chromophobe; KIRC, kidney renal clear cell carcinoma; KIRP, kidney renal papillary cell carcinoma; LIHC, liver hepatocellular carcinoma; LUAD, lung adenocarcinoma; LUSC, lung squamous cell carcinoma; PRAD, prostate adenocarcinoma; READ, rectum adenocarcinoma; STAD, stomach adenocarcinoma; THCA, thyroid carcinoma; UCEC, uterine corpus endometrial carcinoma.. ^*^Adjusted p < 0.05, ^**^Adjusted p < 0.01, ^***^Adjusted p < 0.001.

To understand the role of VEGF family genes in human cancers, we examined their expression patterns across 33 cancers. *VEGFB* has the highest gene expression level, and *VEGFD* has the lowest gene expression level (Fig. 1A). All expression pairs of VEGF family genes showed positive correlations, suggesting potential common features in biological functions and structures (Fig. 1B). The strongest positive correlation was observed between *VEGFC* and *PGF* (r = 0.34). Differential expression analysis of each VEGF family gene was performed in 18 cancers which have more than five normal samples. A higher expression of VEGF family genes, except *VEGFD*, was observed in tumor tissues than in normal tissues across most cancers (Fig. 1C–H). Notably, up-regulated *VEGFA* and *PGF* were observed in 15 cancers, with the highest differences observed in kidney renal clear cell carcinoma (KIRC) (Fig. 1C). The expression of *VEGFD* tended to be down-regulated in most cancers, except Cholangiocarcinoma (CHOL) and liver hepatocellular carcinoma (LIHC). Moreover, *VEGFA*, *VEGFB*, *VEGFC*, and *PGF* expression was significantly increased in CHOL, head and neck squamous cell carcinoma (HNSC), KIRC, and LIHC tumor tissues. LIHC was the only cancer with significant up-regulation of all VEGF family genes.

### Clinical correlation of VEGF family genes

To predict whether the expression of VEGF family genes promotes or inhibits cancer, we performed a survival analysis in 33 cancers. Kaplan-Meier survival curves and univariate Cox proportional hazard ratio were used to investigate the relationship between the expression levels of VEGF family genes and patients' overall survival (Supplementary Table 2, Supplementary Fig. 1). Each VEGF family gene showed significant associations with cancer prognosis in at least four cancers (p < 0.05).

The expression of *VEGFA*, *VEGFC*, and *PGF* showed significant associations with poor prognosis in six different cancers (p < 0.05) (Supplementary Fig. 1): *VEGFA* played a damaging role in cervical squamous cell carcinoma and endocervical adenocarcinoma (CESC), colon adenocarcinoma (COAD), kidney renal papillary cell carcinoma (KIRP), brain lower grade glioma (LGG), LIHC, and prostate adenocarcinoma (PRAD); *VEGFC* played a damaging role in HNSC, LGG, lung adenocarcinoma (LUAD), lung squamous cell carcinoma (LUSC), mesothelioma (MESO), and stomach adenocarcinoma (STAD); and *PGF* played a damaging role in adrenocortical carcinoma (ACC), KIRP, LIHC, Rectum adenocarcinoma (READ), STAD, and uveal melanoma (UVM). By contrast, the expression of *VEGFB* and *VEGFD* was related to a better prognosis across the three cancers: *VEGFB* was a favorable prognostic factor for esophageal carcinoma (ESCA), pancreatic adenocarcinoma (PAAD), and sarcoma (SARC); and *VEGFD* was a favorable prognostic factor for LUAD, PAAD, and SARC.

Significant prognostic differences were observed in LGG based on the expression levels of three VEGF family genes. In LGG, high expression of *VEGFA* and *VEGFC* was significantly associated with worse prognosis, while high *PGF* expression was significantly associated with a better prognosis.

**Fig. 2.**
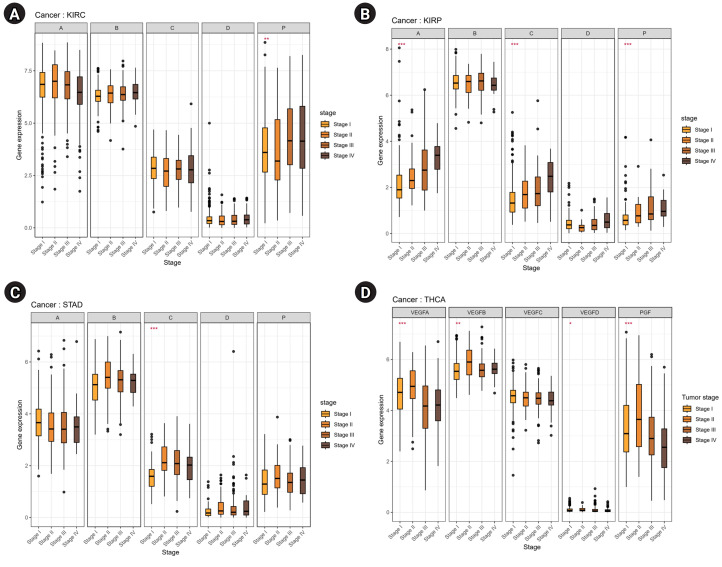
Correlations of vascular endothelial growth factor (VEGF) family genes with patients’ overall survival and tumor stages. Only cancers with p < 0.05 in at least three VEGF family genes are displayed: (A) kidney renal clear cell carcinoma (KIRC), (B) kidney renal papillary cell carcinoma (KIRP), (C) stomach adenocarcinoma (STAD), and (D) thyroid carcinoma (THCA). ^*^Adjusted p < 0.05, ^**^Adjusted p < 0.01, ^***^Adjusted p < 0.001.

Moreover, we analyzed the relationship between the tumor stage and the expression of VEGF family genes. Cancers exhibiting a statistically significant difference (adjusted p < 0.05) in at least three VEGF family genes are shown in Fig. 2. In KIRC, the expression of *PGF* was significantly associated with the tumor stage (Fig. 2A). In KIRP, *VEGFA*, *VEGFC*, and *PGF* were differentially expressed across tumor stages. Except for *VEGFB* and *VEGFD*, the genes showed an increasing tendency in gene expression from stages I to IV in KIRP (Fig. 2B). In STAD, the expression of *VEGFB*, *VEGFC*, and *PGF* showed the highest expression in stage II (Fig. 2C). In Thyroid carcinoma (THCA), most of the VEGF family genes, except *VEGFC*, were differentially expressed in different tumor stages (Fig. 2D). Therefore, the expression of VEGF family genes across different tumor stages may be used to estimate potential cancer progression.

### Association with hypoxia of VEGF family genes and its targeting miRNAs

**Fig. 3.**
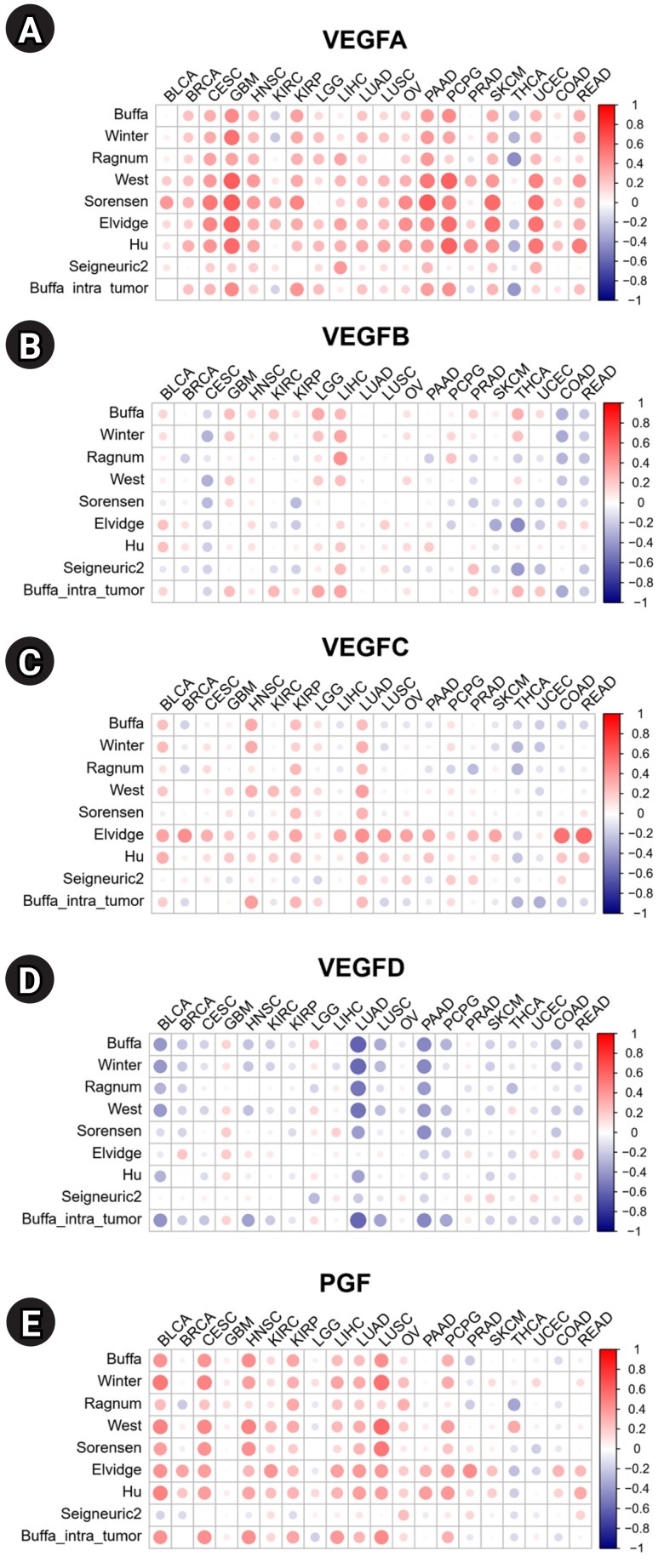
Correlations between hypoxia scores and mRNA expression of vascular endothelial growth factor (VEGF) family genes: (A) *VEGFA*, (B) *VEGFB*, (C) *VEGFC*, (D) *VEGFD*, and (E) placental growth factor *(PGF)*. BLCA, bladder urothelial carcinoma; BRCA, breast invasive carcinoma; CESC, cervical squamous cell carcinoma and endocervical adenocarcinoma; GBM, glioblastoma multiforme; HNSC, head and neck squamous cell carcinoma; KIRC, kidney renal clear cell carcinoma; KIRP, kidney renal papillary cell carcinoma; LGG, brain lower grade glioma; LIHC, liver hepatocellular carcinoma; LUAD, lung adenocarcinoma; LUSC, lung squamous cell carcinoma; OV, ovarian serous cystadenocarcinoma; PAAD, pancreatic adenocarcinoma; PCPG, pheochromocytoma and paraganglioma; PRAD, prostate adenocarcinoma; SKCM, skin cutaneous melanoma; THCA, thyroid carcinoma; UCEC, uterine corpus endometrial carcinoma; COAD, colon adenocarcinoma; READ, rectum adenocarcinoma.

Hypoxia, a precursor for angiogenesis, plays an important role in cancer progression and affects TME [14]. We investigated the correlations between the expression of VEGF family genes and hypoxia scores in 20 cancers (Fig. 3). High expression of *VEGFA* is positively correlated with hypoxia scores in most tumors, indicating a potential correlation with tumor aggressiveness. By contrast, the expression level of *VEGFD* is negatively correlated with the hypoxia score, suggesting *VEGFD* is mainly expressed during normoxia. Overall, *VEGFA* showed strong positive correlations with hypoxia compared to other VEGF family genes (Fig. 3A–E). *VEGFC* showed positive correlations with all hypoxia scores in LUAD (Fig. 3C). *VEGFD* showed strong negative correlations with most of the hypoxia scores in Bladder urothelial carcinoma (BLCA), LUAD, and PAAD (Fig. 3D). *PGF* showed positive correlations with hypoxia scores across most cancers (Fig. 3E).

Correlation analysis was performed to explore the association between the Buffa hypoxia score and the expression of miRNAs targeting VEGF family genes (Supplementary Fig. 2): Hsa-miR-101-3p targeting *VEGFA* and *VEGFC* was the only miRNA that showed a negative correlation with hypoxia in all cancers (Supplementary Fig. 2A and 2C); Hsa-miR-130b-5p targeting *VEGFB* showed positive correlations across most cancers, except for pheochromocytoma and paraganglioma (PCPG) and THCA (Supplementary Fig. 2B); Hsa-miR-940 targeting *VEGFB* was positively correlated with hypoxia in most cancers, except for BLCA and THCA; Hsa-miR-218-5p targeting *VEGFC* showed negative correlations with hypoxia in 18 cancers (Supplementary Fig. 2C); and hsa-miR-335-5p targeting *VEGFD* was positively correlated with hypoxia in 15 cancers (Supplementary Fig. 2D).

### Immune correlation of VEGF family genes

**Fig. 4.**
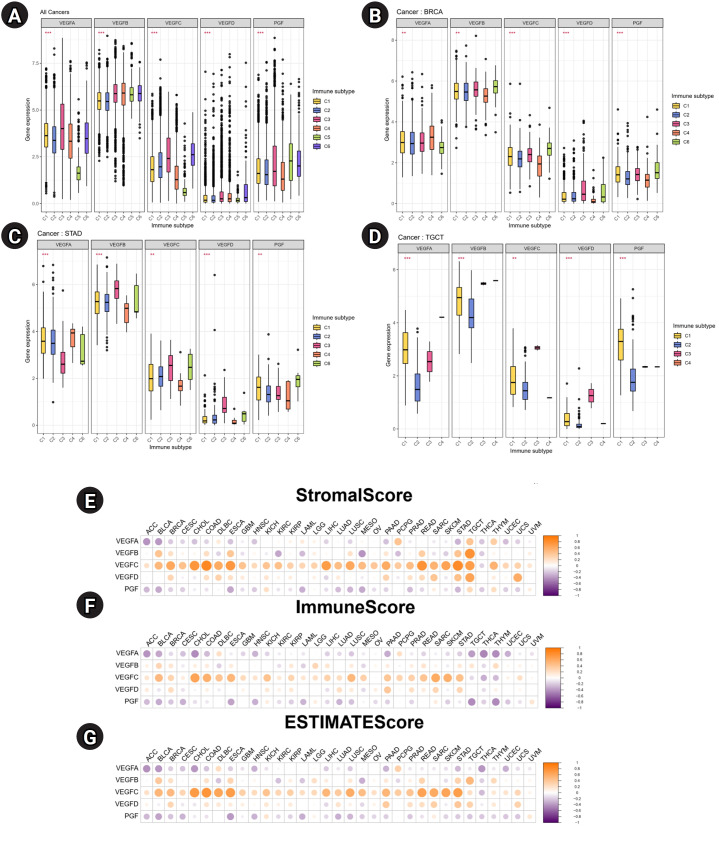
Correlations of vascular endothelial growth factor (VEGF) family genes with immune subtypes, cancer stemness score, and the tumor microenvironment in pan-cancer. (A) A Box plot showing the expression of VEGF family genes differed across pan-cancer. (B-D) The overall transcriptional expression level of VEGF family genes in (B) breast invasive carcinoma (BRCA), (C) stomach adenocarcinoma (STAD) and (D) testicular germ cell tumors (TGCT). *Adjusted p < 0.05; **Adjusted p < 0.01; ***Adjusted p < 0.001. (E-G) Correlation plots demonstrate associations between the mRNA expression of VEGF family genes and (E) Stromal score, (F) Immune score, and (G) ESTIMATE score calculated by the ESTIMATE algorithm. Orange and purple represent a positive and negative correlation, respectively.

To identify the associations between the expression of VEGF family genes and immune components in tumor, correlation analysis was performed. Across multiple cancers, *VEGFB* was highly expressed in six immune subtypes than other VEGF family genes (Fig. 4A). In BRCA, VEGF family genes except *VEGFA* showed high expression in C3 and C6 (Fig. 4B). In STAD, *VEGFD* showed the highest expression in C3 (Fig. 4C). In testicular germ cell tumors (TGCT), all VEGF family genes showed the lowest expression in C2 (Fig. 4D). The TME plays crucial roles in tumor initiation, progression, and metastasis [15], so we investigated the relationship between the expression of VEGF family genes and the scores related to immune infiltration. Notable positive associations between stromal, immune, and ESTIMATE scores and *VEGFC* were observed in most cancers (Fig. 4E–G). *VEGFA* and *PGF* levels were inversely associated with these scores in most cancers.

### Drug sensitivity of VEGF family genes

To investigate the relationship between the responsiveness of more than 200 chemotherapy drugs in NCI60 cancer cell lines and the expression of VEGF family genes, correlation analysis was conducted (p < 0.01) (Supplementary Fig. 3). *VEGFA* expression was strongly and positively correlated with sensitivity to SAR-125844 (r = 0.66), abiraterone (r = 0.56), BLU-667 (r = 0.56), AZD-3229 (r = 0.56), and itraconazole (r = 0.46) (Supplementary Fig. 3A). *VEGFC* expression was distinctively correlated with good responses to JNJ-3387618 (r = 0.65), JNJ-38877605 (r = 0.57), staurosporine (r = 0.55), BLU-667 (r = 0.51), momelotinib (r = 0.51), dimethylfasudil (r = 0.5), zoledronate (r = 0.47), telatinib (r = 0.48), and dastinib (r = 0.47) (Supplementary Fig. 3B). 7–Hydroxystaurosporine showed a negative correlation with *VEGFC* expression (r = –0.35).

## Discussion

In this study, we aimed to explore the potential oncogenic roles of VEGF family genes across pan-cancer through sophisticated bioinformatic analyses. We observed heterogeneity in the expression patterns of VEGF family genes across different cancers. Except for CHOL and LIHC, *VEGFD* was down-regulated in 16 cancers. However, *VEGFA* and *PGF* were up-regulated in tumor tissues across most cancers including BRCA and HNSC (p < 0.001). The literature has reported that *VEGFA* expression was significantly increased in breast cancer and head and neck cancer [16,17].

In survival analysis, the expression of VEGF family genes was closely related to patients' survival; thus, these genes may be potential clinical prognostic indicators. Our findings showed the associations between the high expression of *VEGFA*, *VEGFC*, and *PGF* and poor prognosis across various cancers, suggesting that they may serve as risk factors for tumor progression. Increased expression of *VEGFA* and *PGF* was linked to poor prognosis in KIRP and LIHC. There is evidence that serum *VEGFA* levels are associated with poor prognosis in LIHC [18]. In addition, increased *VEGFA* and *VEGFC* were linked to poor prognosis in LGG. Another study revealed that inhibition of *VEGFA* prolongs the survival of patients with glioblastoma [19]. Furthermore, high expression of *VEGFC* and *PGF* was correlated with poor prognosis in STAD, supported by Li and Han [20]. We demo*nst*rated an association between expression of *VEGFB* and *VEGFD* and favorable prognoses in multiple cancers, including PAAD and SARC. Notably, we are the first to demo*nst*rate these results. Across tumor stages, *VEGFA*, *VEGFC*, and *PGF* exhibited an increasing expression tendency as the tumor stage advanced. In STAD, these genes showed a lower expression in stage I than in the other stages. Because the stage number indicates the extent of cancer spread, these results suggest that the expression of these genes is a clinicopathological marker.

Our investigation of this correlation with hypoxia provides insights into the potential role of VEGF family genes in driving malignant processes. For 20 cancers, the expression of *VEGFA* was positively associated, except for KIRC, PRAD, and THCA. This finding is supported by the literature indicating that hypoxia induces *VEGFA* expression [21]. Moreover, *PGF* exhibits a strong positive correlation with hypoxia in many cancers, including ovarian serous cystadenocarcinoma (OV). Previous study reported that epithelial ovarian cancer patients with high *PGF* expression show poor chemotherapy response and unfavorable prognosis [22]. We observed a distinct negative correlation between *VEGFD* expression and hypoxia across most cancers, but no study has established this finding.

Furthermore, we investigated the correlation between hypoxia scores and miRNAs targeting VEGF family genes. Hsa-miR-101-3p targeting *VEGFA* showed a negative correlation with hypoxia scores across most cancers, including LUAD and LUSC, suggesting its role as a tumor suppressor. This finding was supported by a finding in the literature that hsa-miR-101-3p inhibited *VEGFA* expression, which mediates invasion of lung cancer cells [23]. Hsa-miR-101-3p, which also targets *VEGFC*, was negatively correlated with hypoxia in LIHC. Another study revealed that hsa-miR-101-3p inhibited cell migration by suppressing *VEGFC* expression in hepatocellular carcinoma [24]. Moreover, hsa-miR-130b-5p targeting *VEGFB* exhibited a positive correlation with hypoxia in various cancers including BRCA and LUAD. Its involvement in hypoxia by regulating *VEGFB* has not been demo*nst*rated. Consistent with the positive association between hsa-miR-940 expression and the hypoxia score shown in this study, hsa-miR-940 overexpression was correlated with tumor progression in gastric cancer, pancreatic carcinoma, and ovarian cancer [25-27]. We observed a negative correlation between hsa-miR-218-5p expression and hypoxia score in LUAD. Another study demo*nst*rated that enhanced expression of hsa-miR-218-5p inhibited cell viability and migration in LUAD [28].

We observed that VEGF family genes were significantly correlated with the immune subtypes. In STAD, the expression of *VEGFC* and *PGF* was high in aggressive immune subtype C6 (TGF-β dominant), which is related to poor prognosis. These findings are consistent with our survival results, and the results of another study demo*nst*rating that hypoxic tumor cells promote TGF-β activation, which has a tumor-promoting effect [29]. A consistently high expression of *VEGFC* in stromal, immune, and ESTIMATE scores indicated lower tumor purity. We observed that *VEGFC* was associated with poor prognosis across multiple cancers. Studies have demo*nst*rated an association between low tumor purity and poor prognosis across various cancers [30,31]. The association between the VEGF family genes and drug response is also notable. Increased expression of *VEGFA* and *VEGFC* enhances sensitivity to various chemotherapeutic drugs.

Using integrated bioinformatics analyses, we comprehensively overviewed the roles of VEGF family genes in tumor progression and hypoxia. Because our findings were not validated by independent datasets, further validation using *in vitro* and *in vivo* experiments is necessary. The dynamics of gene families vary widely, so interpreting the overall pattern of positive correlations requires caution. Also, some VEGF family genes are included in multiple hypoxia signatures, which could lead to a bias in the correlation between hypoxia scores and expression level of VEGF family genes. Considering that the hypoxia scores were influenced by the expression levels of not only *VEGFA* but also other hypoxia-related genes, investigating the comprehensive relationship between the hypoxia scores and the VEGF family genes is not devoid of significance.

Overall, *VEGFD* exhibits opposite trend compared to other VEGF family genes. Thus, further research is needed to elucidate the directionality of *VEGFD*'s behavior and its potential implications in hypoxia and other carcinogenic mechanisms.

In this study, we systematically investigated the expression of VEGF family genes and their relationships with patients' survival, hypoxic status, immune subtypes, and drug response in a pan-cancer analysis. Although our findings require further validation from laboratory results, we have provided a detailed overview of the biological functions of VEGF family genes in hypoxia. Thus, we provide insights into the role of VEGF family genes in cancers and provide blueprints for further research on their role in hypoxic TME.

## Supplementary Materials

Supplementary Table 1. The Cancer Genome Atlas (TCGA) pan-cancer data and abbreviations for the studied cancer types

Supplementary Table 2. Univariate cox proportional hazard ratios of VEGF family genes according to gene expression across 33 cancers

Supplementary Fig. 1. Kaplan-Meier survival curves comparing high and low expressions of vascular endothelial growth factor (VEGF) family genes (p < 0.05). Overall survival by *VEGFA* expression in different cancers: BLCA, CESC, COAD, KIRP, LGG, LIHC, and PRAD. Overall survival by *VEGFB* expression in different cancers: COAD, ESCA, PAAD, and SARC. Overall survival by *VEGFC* expression in different cancers: HNSC, LGG, LUAD, LUSC, MESO, PCPG, STAD, and UCEC. Overall survival by *VEGFD* expression in different cancers: LUAD, LIHC, PAAD, SARC, and THYM. Overall survival by *PGF* expression in different cancers: ACC, KIRP, LGG, LIHC, PRAD, READ, STAD, and UVM.

Supplementary Fig. 2. Correlation between Buffa hypoxia score and the expression of miRNAs targeting each vascular endothelial growth factor (VEGF) family gene. miRNAs targeting (A) *VEGFA*, (B) *VEGFB*, (C) *VEGFC*, and (D) *PGF*.

Supplementary Fig. 3. Correlation between drug response and vascular endothelial growth factor (VEGF) family genes in pan-cancer. (A) Correlations between *VEGFA* expression and sensitivity of SAR-125844, Abiraterone, BLU-667, AZD-3229, and itraconazole. (B) Correlations between *VEGFC* expression and sensitivity of JNJ-3887618, JNJ-38877605, staurosporine, BLU-667, momelotinib, dimethylfasudil, zoledronate, telatinib, dastinib, and 7-hydroxystaurosporine.
