# Supplementary material for: Unraveling the hypoxia modulating potential of VEGF family genes in pan-cancer
Source: Genomics Inform. 2023 Sep 27;21(4):e44. doi: 10.5808/gi.23061 (PMC10788353; doi:10.5808/gi.23061)

Supplementary Fig. 1. Kaplan-Meier survival curves comparing high and low expressions of VEGF family genes ($p < 0.05$).

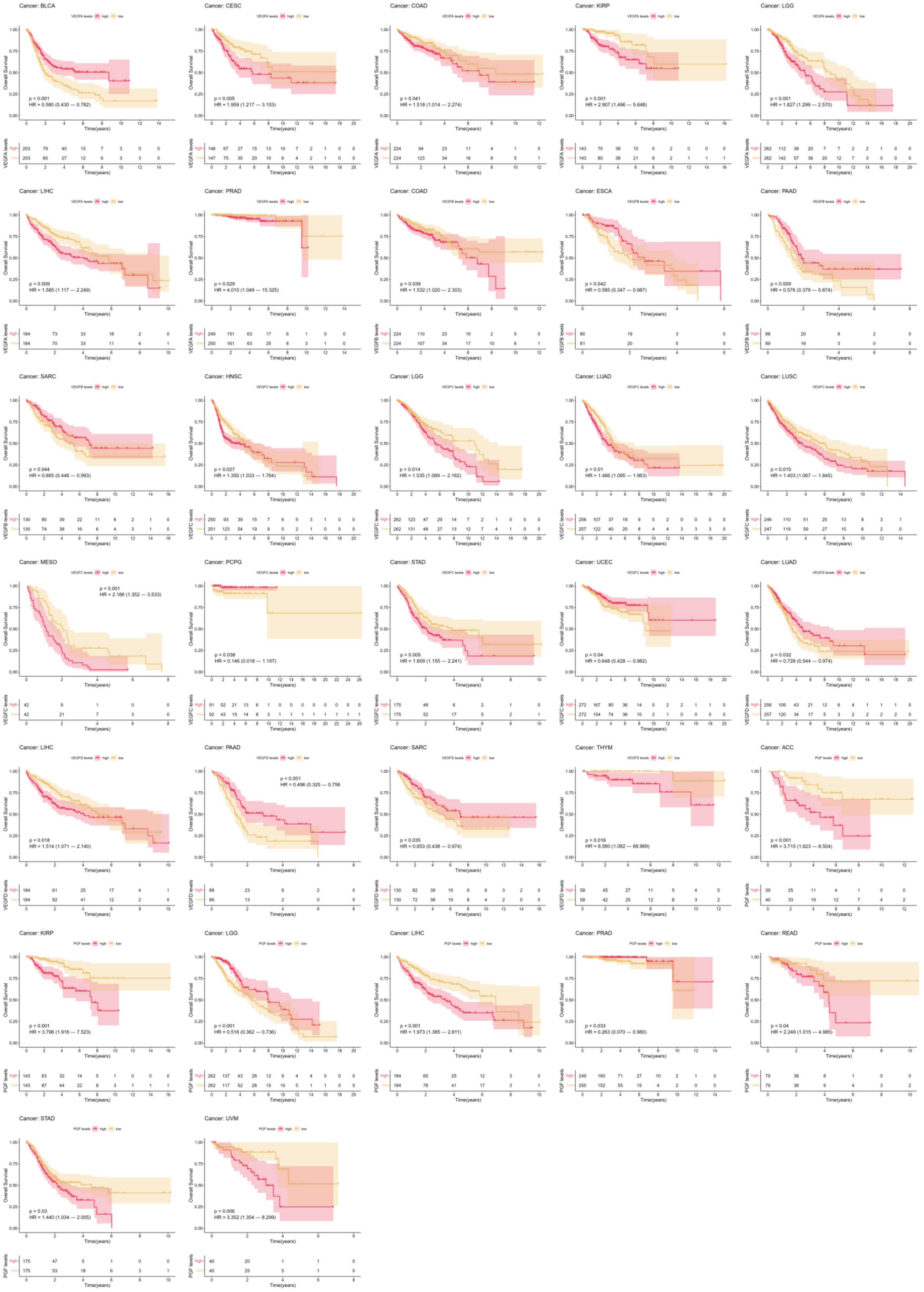

Supplement: Supplementary Fig. 1. — Kaplan-Meier survival curves comparing high and low expressions of vascular endothelial growth factor (VEGF) family genes (p < 0.05). Overall survival by VEGFA expression in different cancers: BLCA, CESC, COAD, KIRP, LGG, LIHC, and PRAD. Overall survival by VEGFB expression in different cancers: COAD, ESCA, PAAD, and SARC. Overall survival by VEGFC expression in different cancers: HNSC, LGG, LUAD, LUSC, MESO, PCPG, STAD, and UCEC. Overall survival by VEGFD expression in different cancers: LUAD, LIHC, PAAD, SARC, and THYM. Overall survival by PGF expression in different cancers: ACC, KIRP, LGG, LIHC, PRAD, READ, STAD, and UVM. [file gi-23061-Supplementary-Fig-1.pdf]
