# Supplementary material for: Unraveling the hypoxia modulating potential of VEGF family genes in pan-cancer
Source: Genomics Inform. 2023 Sep 27;21(4):e44. doi: 10.5808/gi.23061 (PMC10788353; doi:10.5808/gi.23061)

Supplementary Fig. 2. Correlation between Buffa hypoxia score and the expression of miRNAs targeting each VEGF family gene.

miRNAs targeting (A) *VEGFA*, (B) *VEGFB*, (C) *VEGFC*, and (D) *PGF*.

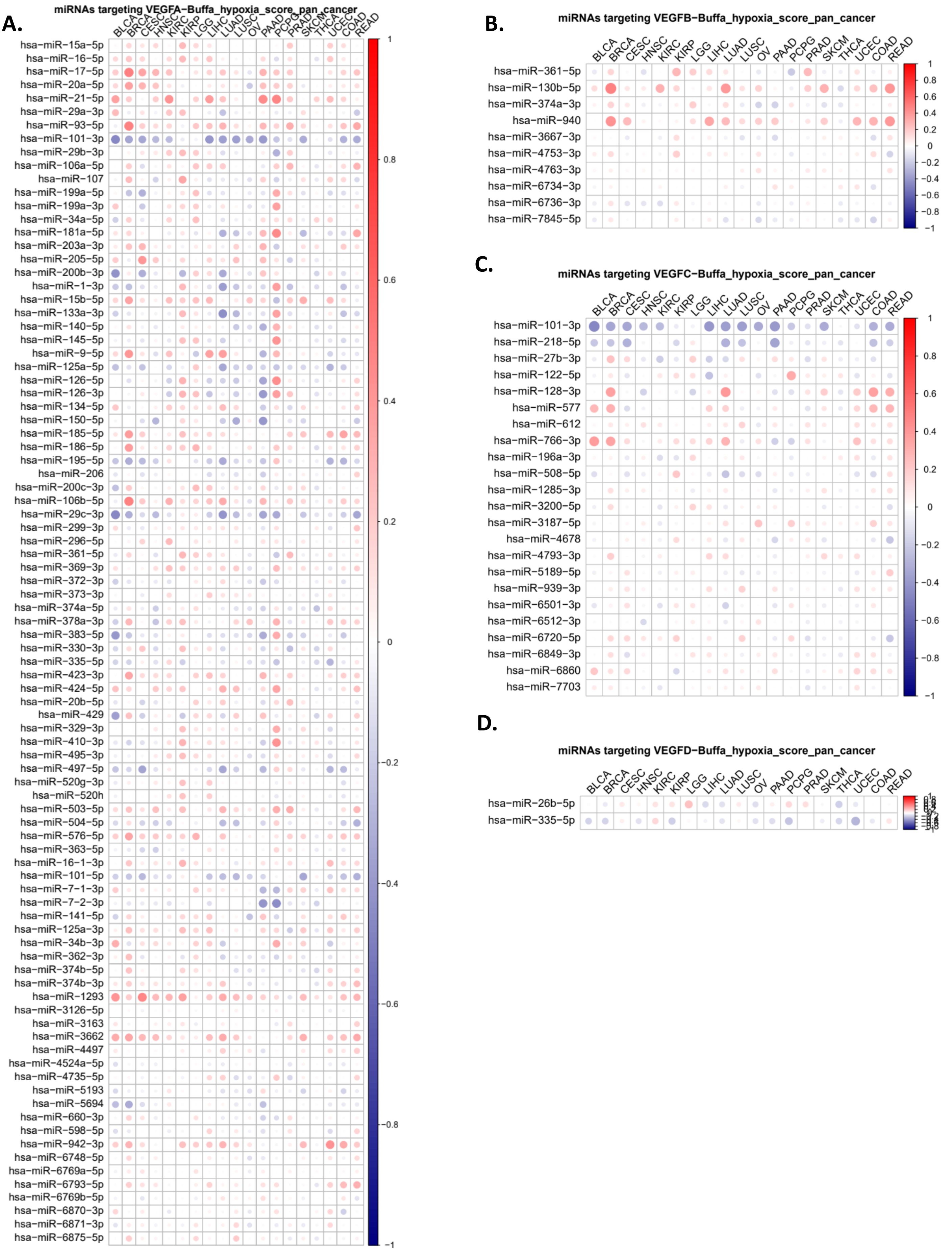

Supplement: Supplementary Fig. 2. — Correlation between Buffa hypoxia score and the expression of miRNAs targeting each vascular endothelial growth factor (VEGF) family gene. miRNAs targeting (A) VEGFA, (B) VEGFB, (C) VEGFC, and (D) PGF. [file gi-23061-Supplementary-Fig-2.pdf]
