# Supplementary material for: Unraveling the hypoxia modulating potential of VEGF family genes in pan-cancer
Source: Genomics Inform. 2023 Sep 27;21(4):e44. doi: 10.5808/gi.23061 (PMC10788353; doi:10.5808/gi.23061)

Supplementary Fig. 3. Correlation between drug response and VEGF family genes (p < 0.05).
(A) *VEGFA* and (B) *VEGFC*.

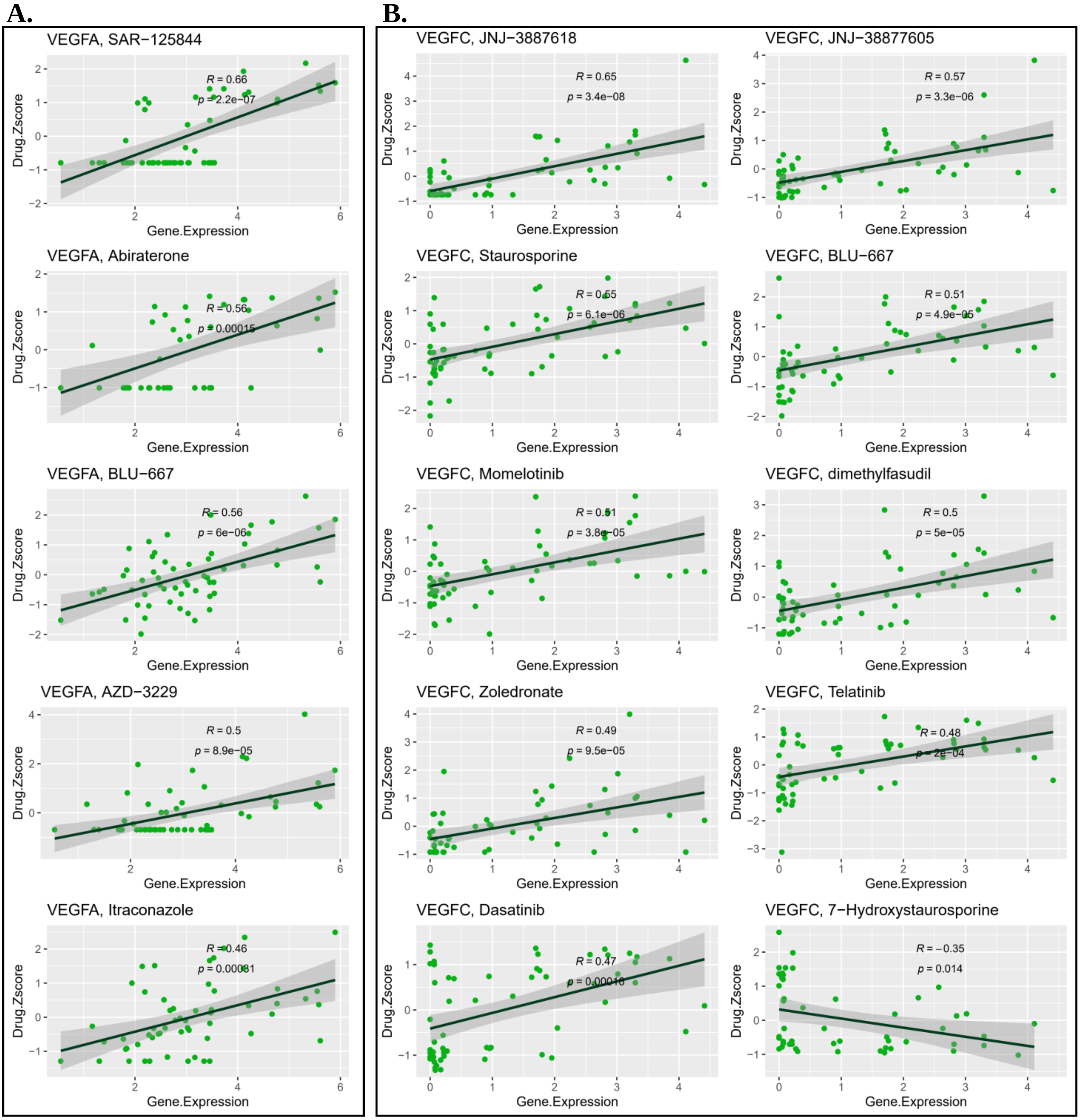

Supplement: Supplementary Fig. 3. — Correlation between drug response and vascular endothelial growth factor (VEGF) family genes in pan-cancer. (A) Correlations between VEGFA expression and sensitivity of SAR-125844, Abiraterone, BLU-667, AZD-3229, and itraconazole. (B) Correlations between VEGFC expression and sensitivity of JNJ-3887618, JNJ-38877605, staurosporine, BLU-667, momelotinib, dimethylfasudil, zoledronate, telatinib, dastinib, and 7-hydroxystaurosporine. [file gi-23061-Supplementary-Fig-3.pdf]
